# Computational–Experimental Characterization of Transcranial Magneto-Acoustic Stimulation for Dose-Efficient Neural Activation

**DOI:** 10.3390/brainsci16090973

**Published:** 2026-09-14

**Authors:** Ruxin Tan, Fangxuan Chu, Xin Wang, Xiaoqing Zhou, Ren Ma, Tao Yin, Haojun Fan, Zhipeng Liu

**Affiliations:** 1 School of Disaster and Emergency Medicine, Tianjin University, Tianjin 300072, China; tanruxin@tju.edu.cn; 2 Institute of Biomedical Engineering, Chinese Academy of Medical Sciences and Peking Union Medical College, Tianjin 300192, China; 3 Tianjin Institutes of Health Science, Tianjin 300192, China

**Keywords:** computational modeling, neuromodulation, neuronal excitability, transcranial magneto-acoustic stimulation, transcranial ultrasound stimulation

## Abstract

**Highlights:**

**What are the main findings?**

- Compared with transcranial ultrasound stimulation, transcranial magneto-acoustic stimulation reduced the acoustic ED50 by 51.5–76.3% across excitatory and inhibitory neuronal models, shortened firing latency, and promoted transitions from single-spike to burst firing.
- Under matched near-threshold conditions, in vitro calcium imaging and in vivo hippocampal fiber photometry showed larger calcium responses following transcranial magneto-acoustic stimulation, accompanied by increased c-Fos and MAP2 immunoreactivity.

**What are the implications of the main findings?**

- Magneto-acoustic coupling may enable neuronal recruitment at a lower acoustic dose while providing improved temporal control compared with ultrasound stimulation alone.
- The identified cell-type- and frequency-dependent response patterns provide a quantitative basis for optimizing stimulation parameters in precision neuromodulation.

**Abstract:**

**Background/Objectives**: Transcranial magneto-acoustic stimulation (TMAS) is an emerging multiphysics neuromodulation modality that introduces magneto-acoustically induced currents into ultrasound-based neural stimulation. However, how this coupled physical input is converted into neuronal recruitment and measurable biological responses remains insufficiently understood. This study aimed to establish a computational–experimental framework for quantifying TMAS-induced neural activation across stimulation dose, spike timing, and calcium-related response domains. **Methods**: The model combined ultrasound-induced membrane mechanics, charge-based neuronal electrophysiology, pressure-dependent calcium-current modulation, and a Lorentz-force-mediated current source for TMAS. Regular-spiking excitatory neurons and low-threshold-spiking inhibitory interneurons were simulated to estimate excitation thresholds, firing latency, and spike-pattern transitions. Two model-informed stimulation conditions were examined using in vitro calcium imaging, in vivo hippocampal fiber photometry, and c-Fos/microtubule-associated protein 2 (MAP2) immunofluorescence. **Results**: TMAS reduced the half-maximal effective dose relative to transcranial ultrasound stimulation by 71.4–76.3% in regular spiking neurons and 51.5–58.2% in low-threshold spiking interneurons, while shortening firing latency and expanding burst-response domains. Experimentally, near-threshold TMAS produced larger peak calcium responses and greater c-Fos/MAP2 immunoreactivity than TUS. **Conclusions**: TMAS enhances neuronal responsiveness more efficiently than TUS by reducing excitation thresholds, improving spike-timing responses, and reshaping firing-pattern transitions. Consistent with the overall computational comparison, experimental measurements showed greater neuronal activation under TMAS than under TUS at matched ultrasound parameters.

## 1. Introduction

Non-invasive deep brain neuromodulation represents a transformative frontier in neuroscience, offering the potential to treat intricate neurological and psychiatric disorders without the risks associated with surgical intervention. Among current strategies, transcranial ultrasound stimulation (TUS) has emerged as a highly promising modality, capable of penetrating deep brain structures with millimeter-scale spatial resolution [1,2,3]. However, further reducing the required acoustic dose while maintaining robust neuromodulatory efficacy remains a key objective to optimize energy efficiency and broaden its clinical applicability. To overcome this energy-efficiency bottleneck, Transcranial magneto-acoustic stimulation (TMAS) has been recently developed. As an innovative multi-physics approach, TMAS combines ultrasonic waves with a static magnetic field. The movement of ions driven by the acoustic wave within the magnetic field generates a coupled intracranial electric field via the Lorentz force, theoretically enabling a highly efficient dual mechanical–electromagnetic modulation [4,5,6,7,8].

Emerging macroscopic and behavioral evidence consistently highlights the technological superiority of TMAS over traditional TUS. Across both healthy subjects and various disease models, TMAS has demonstrated augmented neuromodulatory efficacy. For instance, recent studies have shown that TMAS elicits stronger electromyographic responses, increases local field potential amplitudes, and more effectively enhances synaptic plasticity and cognition-related neural oscillatory coupling [9,10,11]. Furthermore, in pathological models such as Parkinson’s and Alzheimer’s diseases, TMAS exhibits pronounced neuroprotective effects, including accelerated pathological protein clearance and reduced neuroinflammation [12,13,14]. Despite these promising systemic outcomes, the fundamental biophysical mechanism—specifically how the acoustic mechanical forces and the coupled electromagnetic field jointly interact at the single-neuron and ion-channel levels—remains largely elusive. This mechanistic ambiguity currently limits targeted parameter optimization and clinical translation.

The clinical translation and efficacy optimization of TMAS are fundamentally constrained by a highly coupled, multi-dimensional parameter space. Even in conventional ultrasound neuromodulation, acoustic variables, specifically peak pressure, pulse repetition frequency (PRF), and duty cycle, dictate a delicate balance between mechanical stimulation and thermal accumulation [15,16]. The complexity of optimizing these parameters is heavily underscored by cross-species evidence. Prolonging stimulation duration in the rodent motor cortex, for example, increases the probability of activation without scaling the overall response magnitude [17]. Similarly, behavioral responses in Caenorhabditis elegans peak at PRFs between 30 and 100 Hz [18], whereas rodent electrophysiological data demonstrate that PRF variations can selectively recruit glutamatergic neurons [19]. These species- and region-specific discrepancies strongly indicate that the inherent heterogeneity of ion channels across distinct neuronal subtypes ultimately determines the modulatory outcome [20,21]. Building upon this intricate acoustic foundation, TMAS introduces additional variables such as static magnetic field strength and spatial tissue conductivity gradients. The inclusion of these magneto-acoustic factors exponentially expands the mechanical–electromagnetic parameter space into a high-dimensional and strongly nonlinear regime. Consequently, navigating this landscape to identify safe and effective parameter combinations through conventional trial-and-error animal experimentation is practically unfeasible.

To bridge this critical gap, this study establishes a comprehensive translational framework that integrates biophysical computational modeling with rigorous in vitro and in vivo biological measurements. First, we developed an advanced computational model incorporating ultrasound-induced mechanical stress, dynamic calcium ion fluxes, and the magneto-acoustic coupled electric field to systematically map the parameter spaces for representative excitatory (regular spiking, RS) and inhibitory (low-threshold spiking, LTS) neurons. Crucially, moving beyond purely theoretical predictions, we experimentally assessed overall calcium-associated neuronal activation using in vitro intracellular calcium imaging and in vivo real-time fiber photometry in the mouse hippocampus. Furthermore, we investigated downstream activity-associated molecular responses by profiling the expression of the immediate early gene c-Fos and Microtubule-associated protein 2 (MAP2). By elucidating the coupled mechanisms of TMAS from the physical modeling level down to molecular signaling, this work provides a robust theoretical and biological foundation for energy-efficient, targeted, and spatiotemporally precise neuromodulation in future clinical applications.

## 2. Materials and Methods

### 2.1. Computational Model Development

#### 2.1.1. Membrane Mechanics

In the SONIC model, the nanoscale bilayer structures embedded within the plasma membrane undergo minute displacements (Z) in an ultrasonic field, leading to dynamic changes in the membrane capacitance ($C_{m}$). The relationship between the two can be expressed as:

(1) $$C_{m}\left(z\right)=\frac{C_{m0}\Delta }{a^{2}}\left[Z+\frac{a^{2}-Z^{2}-Z\cdot \Delta }{2Z}\ln\left(\frac{2Z+\Delta }{\Delta }\right)\right]$$

where $C_{m}\left(z\right)$ is the specific membrane capacitance at leaflet deflection Z, $C_{m0}$ is the resting specific membrane capacitance, Z is the maximum leaflet deflection from the resting configuration, a is the bilayer-sonophore radius, and Δ is the initial gap between the two membrane leaflets. Periodic membrane capacitance oscillations near resonant structures can induce fluctuations in the membrane potential ($V_{m}$), thereby affecting neuronal activation in a cell-type-dependent manner.

#### 2.1.2. Electrical Response

To reduce computational stiffness, the mechanical and electrical components were decoupled using an update time step of (Tup = 50 µs). The electrical part of the HH equations was reformulated in terms of the smoother membrane charge $Q_{m}$:

(2) $$Q_{m}\left(t\right)=C_{m}\left(t\right){\cdot V}_{m}\left(t\right)$$

This charge-based form improves numerical stability, and the membrane equation can be written as:

(3) $$\frac{dQ_{m}}{dt}=-\sum I_{ion}{+I}_{stim}$$

where $I_{ion}$ represents the ionic currents through various ion channels, and $I_{stim}$ is the externally applied stimulation current. Under TUS conditions, $I_{stim}$ was set to zero, whereas under TMAS conditions, $I_{stim}$ was defined by the magneto-acoustically induced current.

#### 2.1.3. Ion Channel Model

Regular spiking (RS) excitatory neurons and low-threshold spiking (LTS) inhibitory interneurons were selected to examine cell-type-dependent responses. LTS neurons have a smaller difference between resting potential and firing threshold and possess T-type calcium channels; therefore, both membrane capacitance and calcium current were considered under ultrasound stimulation.

Recent studies [22] have demonstrated that ultrasound can affect neuronal calcium dynamics via calcium-selective mechanosensitive ion channels, such as Piezo1. Zhu et al. [23] further provided regression curves for calcium accumulation induced by ultrasound fields in the cerebral cortex.

Based on these findings, a regulatory factor driven by acoustic pressure P(t) was introduced into the calculation of the T-type calcium current ($I_{ca}$) in the LTS neuron model:

(4) $${I_{ca}}^{\prime }=\left[1.32*10^{-6}*P\left(t\right)+1.5542\right]I_{ca}$$

where P(t) represents the local acoustic pressure, and the coefficient is obtained by linear regression of the data reported by Zhu et al. The experimentally reported pressure-dependent calcium response was used only as a phenomenological scaling factor for the intrinsic T-type calcium current in the LTS model. The robustness of this assumption was evaluated using saturating and no-additional-modulation alternatives (Supplementary Materials). The RS neuron model was constructed according to the literature, incorporating sodium ion channels, delayed rectifier potassium ion channels, M-type potassium ion channels, and leakage currents. In addition to these, the LTS inhibitory interneuron model also includes T-type calcium channels.

#### 2.1.4. TMAS-Induced Coupled Currents

In the TMAS simulations, the vibration velocity of induced charged particles under the influence of Lorentz force is determined by the ultrasonic acoustic pressure.

(5) $$v=\frac{P}{\rho c}$$

where v is the vibration velocity of the charged particles, P is the ultrasound acoustic pressure, ρ is the density of biological tissue, and c is the speed of sound.

The current density in tissue with electrical conductivity σ satisfies

(6) $$J_{TMAS}=\frac{\sigma }{\rho c}BP$$

In the implemented single-compartment model, $J_{TMAS}$ is supplied directly to the membrane charge-balance equation as an equivalent transmembrane current density (A/m^2^).

### 2.2. Simulation Data Analysis

#### 2.2.1. Spike Frequency and Dose–Response Analysis

The primary metric for spike frequency in this study is the mean spike frequency within the stimulation window, computed from the inter-spike intervals during stimulation.

The ultrasound dose is characterized by the spatial peak temporal average intensity (Ispta, unit: W/m^2^). The spatial peak pulse average intensity (Isppa) is approximated as: Isppa = P^2^/(2ρc), where P is the peak acoustic pressure (unit: Pa), ρ is set to 1000 kg/m^3^, and c to 1500 m/s. Ispta is then calculated as Isppa × DC, where DC is the duty cycle. Based on the mean firing rate (FR), a binary response variable is constructed using a 1 Hz threshold. Observations with missing values (NaN) are excluded. For each PRF condition (10, 100, 1000 Hz), a generalized linear model (GLM) with a binomial distribution and probit link is used to descriptively fit the binary response variables as a function of Ispta: $P(Y=1|Ispta)=\Phi (\beta_{0}+\beta_{1}\cdot Ispta)$. For continuity with dose–response terminology, the model-derived ED50 is defined as the value of Ispta corresponding to a 50% crossing of the fitted probit curve and is calculated as ED50 = −β_0_/β_1_. This metric summarizes the location of the deterministic activation transition within the sampled parameter landscape. The relative reduction in ED50 between TUS and TMAS is calculated as: $({ED50}_{TUS}-{ED50}_{TMAS})/{ED50}_{TUS}\times 100\%$.

#### 2.2.2. Comparison of Spike Latency

Spike latency is defined as the time interval from stimulation onset to the first spike and is calculated separately under TUS and TMAS conditions. Trials without spike firing are excluded from latency statistics.

#### 2.2.3. Spike Pattern Classification

All spike times (APtimes) within the stimulation interval are assigned to pulse windows according to the pulse repetition frequency (PRF). Spike patterns are classified as: (a) no firing: APtimes is empty; (b) spike: each activated pulse window contains at most one spike; (c) irregular-burst: at least one pulse window contains two or more spikes; (d) full-burst: all pulse windows contain two or more spikes.

### 2.3. In Vivo Animal Experiments

Adult male C57BL/6 mice (8–10 weeks old, 22–26 g) were obtained from Beijing HFK BioTechnology Co., Ltd. (Beijing, China). All the mice were housed at an appropriate temperature (23–25 °C) and humidity (50–60%) at the Chinese Academy of Medical Sciences and Peking Union Medical College. All the mice were provided adequate food and water. All experimental protocols were performed in accordance with the regulations of the Animal Care and Ethics Committee of the Chinese Academy of Medical Sciences and Peking Union Medical College (SYXK: 2025-0002). In addition, every effort was made to reduce harm to the mice and to minimise the number of animals used in the study. A total of 20 mice were randomly assigned into 5 groups (CON, TUS-1, TMAS-1, TUS-2, TMAS-2; *n* = 4 per group). Anaesthesia was maintained with 1% isoflurane gas. The experimenter responsible for data acquisition and analysis was blinded to the treatment groups. Each animal was subjected to only one stimulation session to avoid carryover effects.

The two experimental settings were prespecified before data inspection as the first RS-model grid points showing activation (DC = 55%; DC = 54% inactive) and transition to Irregular-Burst (DC = 66%; DC = 65% classified as Spike) at PRF = 10 Hz and P = 41.04 kPa. TUS and TMAS were delivered using an FP-1M focused transducer (IOA-AC, Beijing, China; 1 MHz; focal length, 54 mm), an AFG3252 waveform generator, and an HSA4101 power amplifier. A 1-MHz sinusoidal pulse train was applied at PRF = 10 Hz for 10 s. The focal dimensions were approximately 16.64 × 1.04 mm, and ultrasound gel was used for acoustic coupling. Pressure was calibrated at the nominal focus under free-field conditions using an NH1000 1.0-mm needle hydrophone (Precision Acoustics Ltd., Dorchester, Dorset, UK). For TMAS, an 8 × 8 × 4 cm neodymium magnet was positioned lateral to the head, approximately 5 mm from the target, producing approximately 0.3 T at the target with the magnetic field oriented orthogonally to ultrasound propagation.

### 2.4. In Vitro Experiments

We used the mouse hippocampal neuronal cell line HT-22, which is a widely recognized neuronal model that expresses functional voltage-gated sodium and calcium channels and exhibits quantifiable calcium responses to physical stimuli. The cell line was obtained from the Cell Bank of the Chinese Academy of Sciences (Catalog No. GNM47). HT-22 is a subclone of the HT-4 cell line, which was derived from mouse neuronal tissue immortalized with a temperature-sensitive SV40 T antigen. Cells were used at passages 5–10 to ensure genetic stability. Culture preparation and cultivation: The cells were cultivated in a sterile incubator at 37 °C, in 5% CO_2_ and at a constant pH (pH: 7.2–7.4). HT-22 neurons were grown in DMEM supplemented with 10% FBS and 1% PS. The medium was replaced every 2 days to ensure nutrient availability and the cells were passaged for 2 or 3 days to ensure adequate space for growth. All experiments were performed at DIV 3 after seeding, at which point the cells had reached approximately 70–80% confluence. Fura-2 imaging and immunofluorescence used three and four independent culture batches, respectively; individual cells and fields were treated as nested observations.

### 2.5. Live-Cell Calcium Imaging

To experimentally assess the computationally predicted enhancement of neuronal excitability, cultured neurons were incubated with Ca^2+^ imaging buffer containing 2.5 μM Fura 2 (Invitrogen) and 0.05% Pluronic F 127 (Invitrogen) for 30 min at 37 °C in the dark. The cells were subsequently washed with fresh imaging buffer to eliminate excess dye before TMAS or TUS. Fluorescence images of intracellular Fura 2 were captured at regular intervals using a Nikon Eclipse Ti2 E microscope. Ca^2+^ signals were acquired via dual-wavelength excitation (340/380 nm), and the corresponding emission ratios were computed to quantify intracellular Ca^2+^ concentrations. For each coverslip, 8–10 random fields were quantified, with ≥150 cells analyzed per group across three independent culture batches. The experimenter performing image acquisition and quantification was blinded to treatment groups.

### 2.6. In Vivo Fibre Photometry Recording

Mice were anesthetized and mounted on a stereotaxic frame. A 33-gauge microsyringe (Hamilton Company, Reno, NV, USA) was used to infuse 2.5 µL of rAAV-hSyn-GCaMP6s-WPRE-polyA (BrainVTA Co., Ltd., Wuhan, China) into the hippocampal DG (AP: −2.0 mm, ML: +1.4 mm, DV: 1.5 mm) at a constant rate of 30–50 nl/min. Following viral delivery, unilateral optical fibers (RWD Life Science, Shenzhen, China; O.D. 1.25 mm, core 200 μm, NA 0.39) were implanted into the same DG region and permanently anchored with dental cement for subsequent fiber photometry recordings. Animals with obvious surgical damage were excluded from analysis. Following a three-week recovery and viral expression period, TUS and TMAS interventions were administered. Anaesthesia was maintained with 1% isoflurane gas, and sterile medical ultrasound gel was applied to the skull surface to couple the transducer for efficient energy transmission. After identifying the hippocampal coordinates (AP: 2.0 mm, ML: ±1.4 mm, DV: 1.5 mm), stimulation parameters were matched to the computational boundaries: Parameter Set 1 (Spike boundary; DC = 55%, P = 41.04 kPa) and Parameter Set 2 (Irregular-burst boundary; DC = 66%, P = 41.04 kPa). Calcium-dependent fluorescence transients from the targeted neuronal population were continuously monitored using a fiber photometry system (R810, RWD Life Science, Shenzhen, China), with excitation provided by 470-nm and 410-nm LED light sources. ΔF/F was calculated using the following formula: ΔF/F = (F_470_ − F_410__(fitted))/F_410__(fitted), where F_470_ is the raw 470-nm calcium-dependent fluorescence signal, and F_410__(fitted) is the fitted 410-nm isosbestic reference signal used to correct for calcium-independent fluctuations. This approach effectively removes motion artifacts and photobleaching effects from the calcium signal. Each group was normalized using the ΔF/F value of the CON group. Signals were acquired at a sampling rate of 30 Hz. To correct for photobleaching and motion artifacts, the isosbestic reference signal (410 nm) was used to fit the calcium-dependent signal (470 nm) using a linear regression model. The fitted 410-nm component was then subtracted from the 470-nm signal to obtain the corrected calcium-specific signal. The baseline fluorescence was defined as the mean fluorescence intensity during a 2-s window immediately preceding stimulus onset. For each animal, baseline was calculated from the corrected 470-nm signal during this pre-stimulus window. The smoothing processing algorithm is the moving average algorithm available on MATLAB R2022a (MathWorks, Natick, MA, USA). Calcium transient analysis was performed on an 8-s post-stimulus window, which encompassed the entire evoked calcium response. For each animal, calcium responses from 3 stimulation trials were averaged to generate a single value per animal prior to group-level statistical comparisons.

### 2.7. Immunofluorescence Staining

Cells were fixed with 4% PFA for 15 min at room temperature. The cells were blocked for 60 min in blocking buffer (0.5% Triton X-100 with 10% NGS) at room temperature, and then immersed in primary antibody (MAP2, #78667, 1:200; c-Fos, #2250, CST, 1:1000), at 4 °C for 12 h. On the second day, the cells were washed in PBS and stained with a conjugated fluorescent secondary antibody (A-11029 or A-27040, 1:1000, Thermo Fisher Scientific, Waltham, MA, USA) for 1 h in the dark. Nuclei were counterstained with DAPI (1 µg/mL, Sigma-Aldrich) for 5 min. Coverslips were mounted with anti-fade mounting medium. Cell visualization and data analysis were conducted via microscopy (Olympus, Tokyo, Japan; FV1000, 405 nm, 488 nm and 594 nm) and ImageJ version 1.54g (National Institutes of Health, Bethesda, MD, USA). For each coverslip, 6–8 random fields of view were acquired. The experimenter performing image acquisition and quantification was blinded to the treatment groups. Coverslips were coded prior to imaging, and group allocation was revealed only after quantification was completed. 4 independent culture batches (biological replicates) were used for each experimental condition.

### 2.8. Statistical Analysis

Simulation parameter-grid results were treated as deterministic outputs and analyzed descriptively. Experimental data are presented as mean ± SEM. Independent biological replicates comprised three culture batches per group for calcium imaging (*n* = 3), four culture batches per group for immunofluorescence (*n* = 4), and four mice per group for fiber photometry (*n* = 4). Measurements were averaged within each culture batch or animal before analysis. Group differences were assessed using one-way ANOVA followed by Tukey’s multiple-comparison test. Normality and homogeneity of variance were evaluated using the Shapiro–Wilk and Brown–Forsythe tests, respectively. All tests were two-sided, with (*p* < 0.05) considered significant.

## 3. Results

### 3.1. Enhanced Effects by the Coupled Electric Field

**Figure 1:**
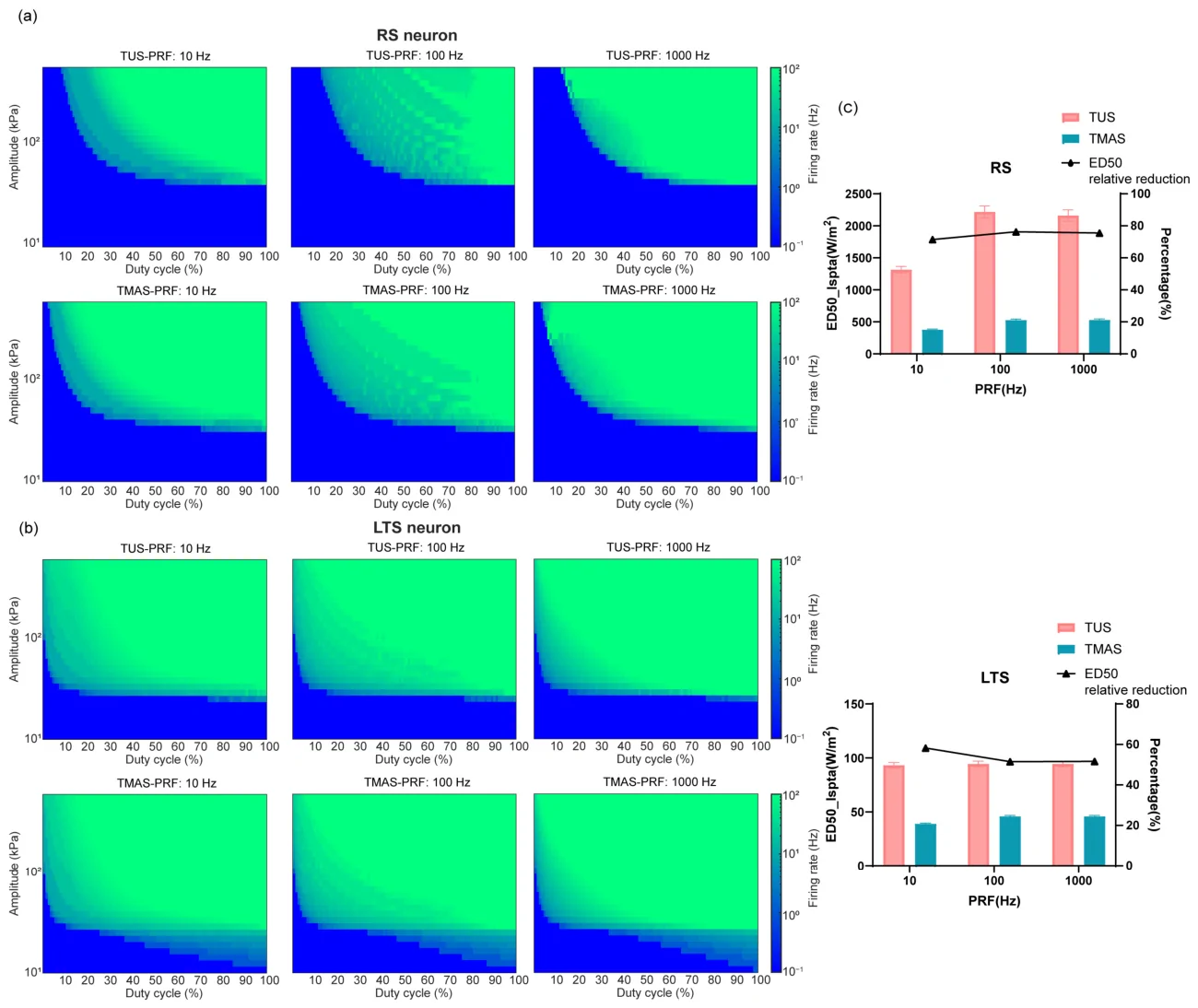
Duty cycle–ultrasound intensity excitation threshold. (**a**) Curve of RS. (**b**) Curve of LTS. (**c**) ED50 excitation thresholds of RS and LTS.

Firstly, the modulatory effect of the coupled electric field in TMAS on the excitation threshold of different neuron types was analyzed. In this simulation, the fundamental ultrasound frequency was set to 1 MHz, and the magnetic field intensity to 0.3 T. The duty cycle ranged from 1% to 100% in increments of 1 percentage point, and the peak acoustic pressure comprised 30 logarithmically spaced values from 10 to 600 kPa. For RS and LTS neurons, firing frequencies were simulated under pulse repetition frequencies of 10 Hz, 100 Hz, and 1 kHz, respectively. The total simulation time was 2 s. Based on the firing frequencies under different parameters after TUS and TMAS, duty cycle–ultrasound intensity excitation threshold curves were plotted (Figure 1).

The threshold curves for duty cycle and ultrasound intensity under TUS and TMAS conditions both demonstrated dependence on duty cycle and ultrasound intensity in the neuron models. At low duty cycles, very high ultrasound intensities were required to evoke neuronal firing; as the duty cycle increased, the required ultrasound intensity gradually decreased, reaching a stable value when the duty cycle exceeded 50%. LTS neurons could be activated by low-intensity ultrasound at much lower duty cycles compared to RS neurons, indicating that LTS neurons required less ultrasound dose than RS neurons. As shown in the figures, under identical ultrasound parameters, the TMAS curve shifted overall to the left relative to the TUS curve, suggesting that the addition of a coupled electric field significantly reduced the ultrasound dose threshold required to trigger spike firing. This phenomenon was observed in 2 neuron types, and for LTS neurons, the required ultrasound intensity continuously decreased as the duty cycle increased.

A further quantitative analysis was conducted to investigate the effect of the coupled electric field on lowering the neuronal excitation threshold (Figure 1c). The results showed that, for RS neurons, compared to TUS, the model-derived ED50 threshold of TMAS was reduced by approximately 71.4% (from 1314.29 W/m^2^ to 375.32 W/m^2^), 76.3% (from 2216.08 W/m^2^ to 526.21 W/m^2^), and 75.6% (from 2159.54 W/m^2^ to 527.90 W/m^2^) at PRFs of 10, 100, and 1000 Hz, respectively. These reductions were significant across all three frequencies, and the trend remained relatively consistent regardless of frequency. For LTS neurons, the ED50 thresholds of TMAS were also consistently lower than those of TUS across all three frequencies: by approximately 58.2% at PRF = 10 Hz (from 93.16 W/m^2^ to 38.94 W/m^2^), 51.5% at PRF = 100 Hz (from 94.48 W/m^2^ to 45.85 W/m^2^), and 51.6% at PRF = 1 kHz (from 94.48 W/m^2^ to 45.77 W/m^2^). The reduction in ED50 was most pronounced at low frequencies and tended to stabilize at higher frequencies.

### 3.2. Enhanced Modulation of Firing Latency by Magneto-Acoustic Coupled Stimulation

**Figure 2:**
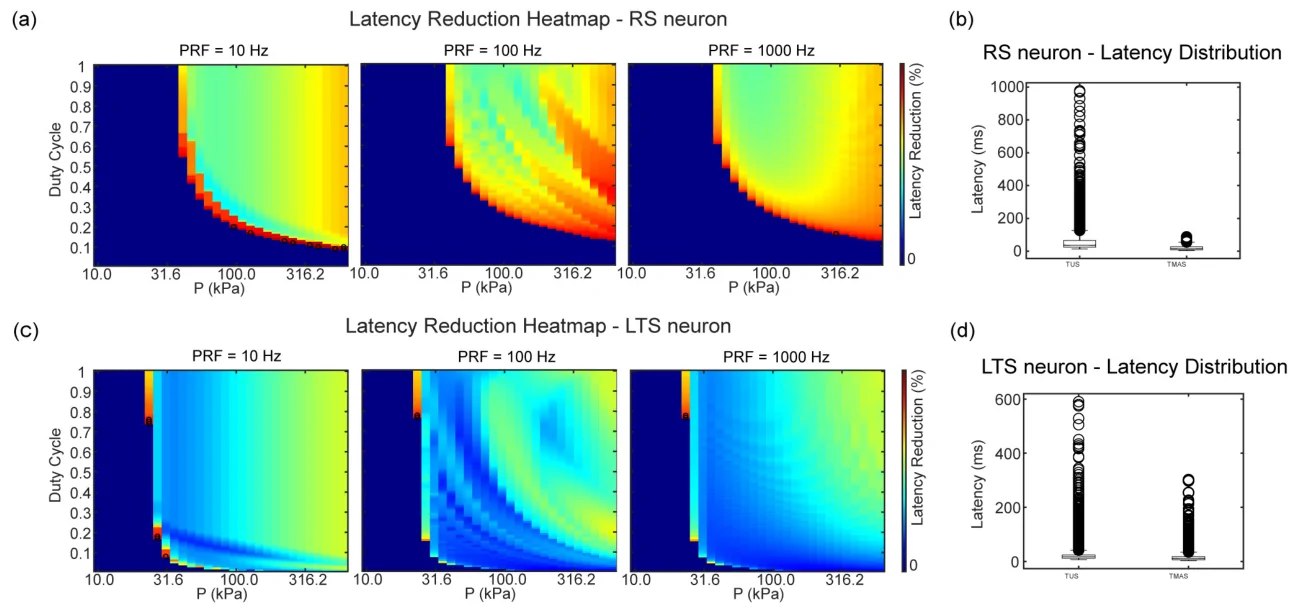
Firing latency reduction under TMAS versus TUS: (**a**) Percentage reduction heatmap for RS. (**b**) Descriptive boxplot for RS. (**c**) Percentage reduction heatmap for LTS. (**d**) Descriptive boxplot for LTS.

Within the parameter space above the threshold for co-triggered firing, we performed a quantitative comparative analysis of the firing latency for RS and LTS neurons. Figure 2a,c presents the distribution of the percentage reduction in latency. The results indicate that, for both RS and LTS neurons, TMAS significantly shortened the firing latency, with the most pronounced effect observed in the parameter regions near the TUS threshold. The top ten parameter points with the highest percentage reduction are highlighted (black circles) in the figures. For RS neurons, 4638 of the 9000 tested parameter combinations produced action potentials under both TUS and TMAS, whereas 4362 conditions were excluded because one or both stimulation modes did not elicit firing. Across the matched co-triggered conditions, TMAS reduced the mean first-spike latency by 41.40 ms relative to TUS. For LTS neurons, 6860 conditions were included and 2140 were excluded, with a mean latency reduction of 9.13 ms under TMAS. Although the overall reduction in LTS neurons was lower than that in RS neurons, in specific high-frequency conditions (PRF = 100 Hz, DC = 0.77, P = 23.33 kPa), the reduction rate reached as high as 90.2%. These findings demonstrate that the superimposed coupled electric field can significantly shorten the firing latency of different neuron types, with the effect being especially prominent near the excitation threshold.

### 3.3. Regulation of Firing Patterns by the Coupled Electric Field

The results demonstrated that TMAS, via its coupled electric field, significantly lowers the neuronal excitation threshold and shortens firing latency. To further clarify its mechanism of action and regulatory effects under near-threshold stimulation, we systematically analyzed the differences in firing patterns of RS and LTS neurons under various pulse repetition frequencies (PRF: 10 Hz, 100 Hz, 1 kHz) and ultrasound intensities. Specifically, the above-threshold parameter space was categorized into three types based on the characteristics of action potential generation within each pulse: Spike (one fixed spike triggered per stimulus pulse), Irregular-burst (≥2 spikes occurring within some pulses, but not all), and Full-burst (≥2 spikes triggered in every pulse). Based on these classifications, heatmaps were generated to show the distribution of the three firing patterns for both TUS and TMAS, and three representative parameter points were selected from each TUS region for further comparison of the firing patterns induced by both stimulation modalities.

**Figure 3:**
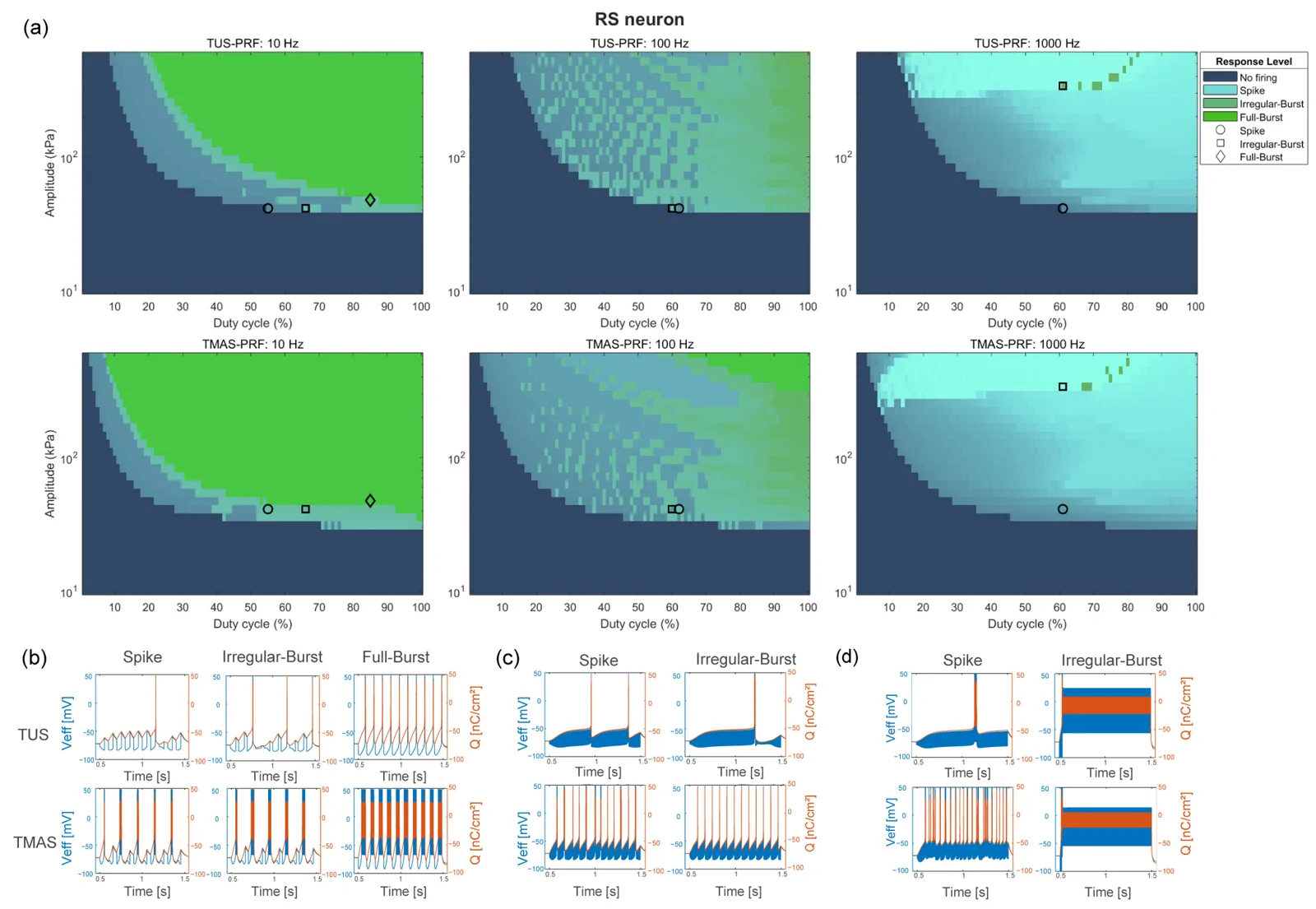
Firing pattern distribution and representative responses of RS. (**a**) Distribution maps of firing patterns (Spike, Irregular-burst, and Full-burst) at PRF = 10 Hz, 100 Hz, and 1 kHz. The top row represents TUS, and the bottom row represents TMAS. (**b**) Representative traces at PRF = 10 Hz. (**c**) Representative traces at PRF = 100 Hz. (**d**) Representative traces at PRF = 1 kHz.

For RS neurons at PRF = 10 Hz, the boundaries among the three firing patterns were distinct, with high ultrasound doses primarily resulting in burst firing (Figure 3a, left panels). As the repetition frequency increased, the duration of each individual stimulus pulse decreased, leading to an expansion of the Spike region at PRF = 100 Hz (Figure 3a, middle panels), and at PRF = 1 kHz, nearly the entire parameter space was occupied by the Spike pattern (Figure 3a, right panels). Under PRF = 10 Hz (Figure 3b), comparison of three representative parameter points revealed that under identical ultrasound conditions, TUS only induced a single spike (with the membrane potential showing obvious cumulative depolarization between pulses, requiring multiple pulse accumulations to elicit a spike), whereas TMAS could directly induce burst firing. Moreover, when TUS required multiple ultrasound pulses to trigger a burst, TMAS could achieve this with fewer pulses, resulting in a higher firing rate within the same timeframe. At parameter points in the Full-burst region, TMAS exhibited both shorter firing latency and higher firing rates compared to TUS. This pattern was also observed at PRF = 100 Hz (Figure 3c) and 1 kHz (Figure 3d), indicating that the coupled electric field in TMAS reduces the ultrasound dose required to trigger bursts, decreases the number of necessary pulses, and accelerates the depolarization accumulation process. However, at high PRF, the Full-burst region disappeared for TUS.

**Figure 4:**
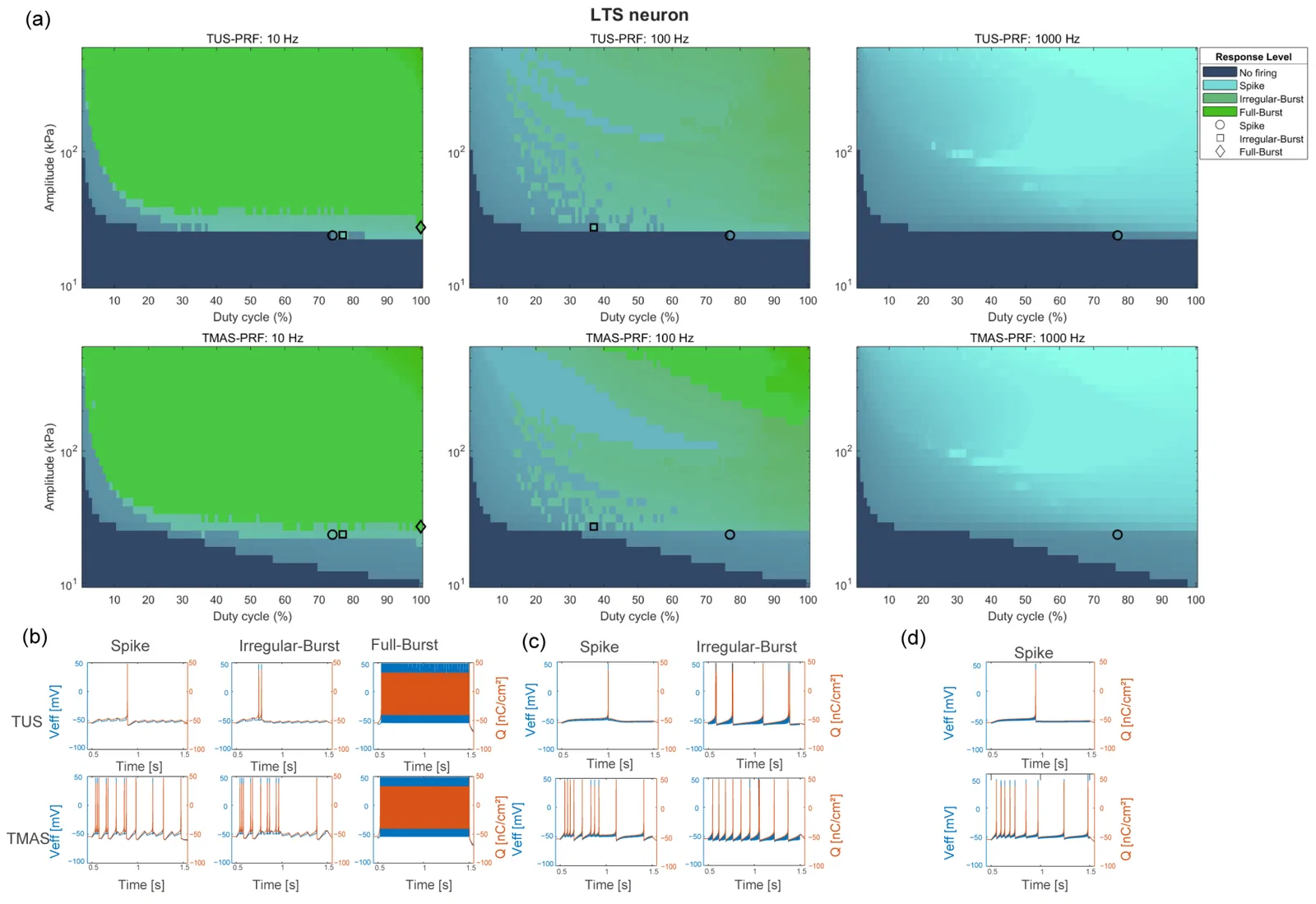
Firing pattern distribution and representative responses of LTS. (**a**) Distribution maps of firing patterns (Spike, Irregular-burst, and Full-burst) at PRF = 10 Hz, 100 Hz, and 1 kHz. The top row represents TUS, and the bottom row represents TMAS. (**b**) Representative traces at PRF = 10 Hz. (**c**) Representative traces at PRF = 100 Hz. (**d**) Representative traces at PRF = 1 kHz.

In LTS neurons, the trends in firing pattern changes induced by TUS and TMAS were similar to those observed in RS neurons (Figure 4). However, under high PRF conditions, both TUS and TMAS only elicited single-spike firing in LTS neurons (Figure 4a, right panels). At PRF = 10 Hz (Figure 4b), due to the lower firing threshold of LTS neurons, increasing the duty cycle by just 3% at the same ultrasound intensity was sufficient to shift the firing pattern induced by TUS from single spike to burst. Additionally, TMAS significantly reduced the threshold required for LTS neurons to switch from spike to burst across all PRF settings, indicating that the addition of a coupled electric field effectively enhances the ultrasound excitability of low-threshold neurons.

### 3.4. Experimental Assessment of Enhanced Neuronal Excitability and Downstream Activation

**Figure 5:**
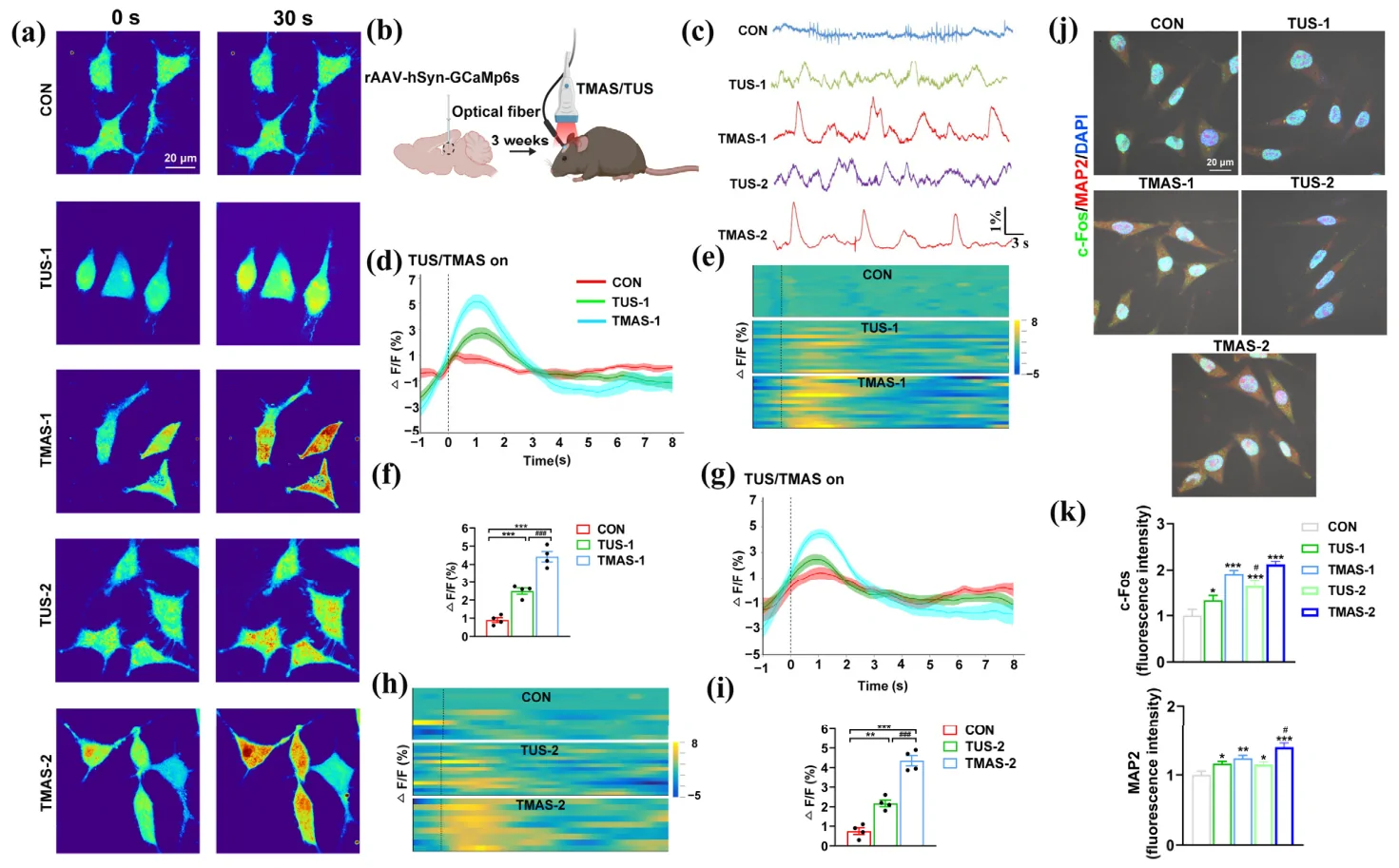
Experimental assessment of calcium-associated neuronal activation and molecular responses. (**a**) Representative pseudocolor images of in vitro intracellular calcium fluorescence. Scale bar, 20 μm. (**b**) Schematic showing hSyn-GCaMp6s virus delivery and fiber implantation in the brain. (**c**) Representative calcium traces. elicited by TUS and TMAS in the brain of mice (**d**,**f**) Averaged calcium transient waveforms (**d**) and quantitative comparison of peak ΔF/F (**f**) under Parameter Set 1 (*** *p* < 0.001, ### *p* < 0.001), *n* = 4 per group. For each mouse, calcium traces were averaged from 3 recording trials. (**e**) Heatmaps depicting the normalized in vivo calcium fluorescence intensity for the CON, TUS-1, and TMAS-1 groups. (**g**,**i**) Averaged calcium transient waveforms (**g**) and quantitative comparison of peak ΔF/F (**i**) under Parameter Set 2 (** *p* < 0.01, *** *p* < 0.001, ### *p* < 0.001). *n* = 4 per group. For each mouse, calcium traces were averaged from 3 recording trials. (**h**) Heatmaps depicting the normalized in vivo calcium fluorescence intensity for the CON, TUS-2, and TMAS-2 groups. (**j**) Representative immunofluorescence images of c-Fos (green), MAP2 (red), and nuclei (DAPI, blue) staining. Scale bar, 20 μm. (**k**) The mean fluorescence intensity of c-Fos and MAP2. *n* = 4 per group. * *p* < 0.05, ** *p* < 0.01, *** *p* < 0.001, # *p* < 0.05.

To experimentally assess whether TMAS enhanced neuronal activation relative to TUS under matched model-informed stimulation conditions, we measured calcium responses in vitro and in vivo. Based on the parameter space mapping from our simulation results (Figure 3), we selected two prespecified discrete-grid transition points specifically for RS neurons at PRF = 10 Hz: Parameter Set 1 for the Spike boundary (DC = 55%, P = 41.04 kPa) and Parameter Set 2 for the Irregular-burst boundary (DC = 66%, P = 41.04 kPa). In vitro, pseudocolor imaging revealed that TMAS induced a markedly greater intracellular calcium influx than TUS under matched parameters (Figure 5a, Supplementary Figure S4). To verify this in vivo, we monitored real-time calcium dynamics in the mouse hippocampal DG region via fiber photometry. Consistent with the in vitro data, fiber photometry revealed that TMAS evoked larger-amplitude calcium transients (Figure 5c,e,h). Quantitative analysis confirmed that peak calcium amplitudes (ΔF/F) were significantly higher in the TMAS groups under both the Spike (Figure 5d,f; *p* < 0.001) and Irregular-burst boundaries (Figure 5g,i; *p* < 0.001). These data strongly corroborate that TMAS crosses the neuronal excitation threshold more efficiently than pure mechanical TUS at the same ultrasound dose.

Furthermore, to investigate the downstream molecular consequences of this enhanced Ca^2+^ influx, we evaluated the expression of c-Fos (an immediate early gene responsive to calcium elevation) and MAP2 (a cytoskeletal protein) in in vitro cultured cells. Following TMAS treatment, nuclear c-Fos expression in MAP2-identified neurons was notably upregulated compared to the TUS and control groups (Figure 5j), consistent with enhanced transcriptional activation following calcium influx. Concurrently, TMAS robustly enhanced overall MAP2 expression, suggesting a potential increase in microtubule-associated protein expression.

Collectively, these findings indicate that TMAS elicited enhanced calcium responses together with activity-associated changes in c-Fos and MAP2 immunoreactivity.

## 4. Discussion

By integrating computational modeling with in vitro and in vivo experiments, this study demonstrates that TMAS modulates neuronal activity more efficiently than pure TUS. Compared with prior TMAS models that primarily focused on membrane mechanics under fixed acoustic conditions, our framework incorporates both dynamic ultrasonic effects on membrane capacitance and ultrasound-driven calcium dynamics and further compares these predictions with multi-level biological measurements. The results show that the magneto-acoustic coupled electric field lowers excitation thresholds, shortens firing latency, and reshapes firing patterns across neuronal subtypes, while calcium imaging and molecular assays consistently support enhanced calcium-associated neuronal activation under TMAS.

For a period, TMAS was regarded as a form of transcranial electrical stimulation because of its physical principles, and the stimulatory role of ultrasound was overlooked [24,25]. With further investigation into TMAS, it was found that the ultrasonic field in TMAS not only drives oscillations of charged particles to generate a coupled electric field but also plays a direct role in the stimulation process [20,26]. Previous studies based on the Hodgkin–Huxley and Hindmarsh–Rose neural models examined how static magnetic field strength, ultrasound intensity, duty cycle, and repetition frequency affect neuronal firing patterns, providing theoretical guidance for TMAS [27,28]. In 2018, building upon the Izhikevich neuron model, the TMAS model was improved by incorporating the effect of ultrasonic mechanical forces on membrane capacitance in the simulation [29]. In that foundational work, the influence of TMAS was simulated under fixed ultrasound parameters, and the acoustic effect on membrane capacitance was modeled as a sinusoidal variation. To further capture the physiological complexity of TMAS, the model developed in our study not only incorporates dynamic ultrasonic mechanical forces on membrane capacitance but also integrates the critical effects of ultrasound-driven calcium ion dynamics.

### 4.1. Enhanced Neuromodulation of Excitability and Spike-Timing Control

Our study found that TMAS, through the combined action of the ultrasonic field and the coupled electric field, significantly lowered the excitation threshold of different neuron types and improved spike-timing control. Simulation results showed that, relative to TUS, TMAS caused a clear leftward shift in the excitation-threshold curves (Figure 1). For high-threshold RS neurons, TMAS significantly reduced ED50 under all PRF conditions (a 71.4–76.3% decrease across 10–1 kHz), indicating frequency-robust activation. The experimental measurements showed a qualitatively consistent enhancement of calcium-associated neuronal activation under the selected TMAS conditions. Under identical near-threshold boundary parameters, TMAS induced stronger intracellular calcium transients (higher peak ΔF/F) than TUS in both in vitro cultured neurons and the intact mouse brain. This amplified biological response directly mirrors the predicted leftward shift in the threshold curves, supporting the conclusion that the coupled electric field functionally amplifies the neurons’ responsiveness to acoustic energy. Consistent with this, previous studies have shown that ultrasound requires approximately six orders of magnitude more energy than electrical stimulation to initiate an action potential [30]. This enhanced efficiency may arise from the coupled electric field’s ability to directly drive the membrane potential. Regardless of the ultrasound pulse frequency, subthreshold depolarization by the electric field accelerates ultrasound-induced opening of sodium channels, thereby reducing reliance on the activation of mechanosensitive channels, such as the Piezo1 or TRPP family [21,31,32]. In contrast, LTS neurons, due to the inherent properties of low-threshold T-type calcium channels [33,34], were particularly sensitive to TMAS under low-frequency conditions (PRF = 10 Hz), with ED50 reduced by 58.2% and stabilizing at 51.5–51.6% as PRF increased. This difference suggests that the low-frequency sensitivity of LTS neurons is related to the slow inactivation kinetics of their calcium channels. Low-frequency stimulation allows more complete recovery from inactivation, thereby enhancing the synergistic effect of the electric field [4]. This finding provides a theoretical basis for parameter optimization targeting specific neuronal subtypes. When modulating LTS-dominant circuits such as thalamocortical oscillations [35,36], low-frequency TMAS could maximize energy efficiency.

Across matched co-triggered parameter combinations, TMAS shortened first-spike latency in both RS and LTS models (Figure 2), consistent with animal experiments reporting shortened electromyographic response latency [10]. This result validated that the effects between the electric field and ultrasound not only lowered activation thresholds but also improved spike-timing control. Notably, the magnitude of latency shortening peaked near the TUS near-threshold regime; for example, in LTS neurons at PRF = 100 Hz with a high duty cycle, the reduction reached 90.2%. This pattern indicates that the coupled electric field acted through a pre-polarization mechanism, bringing the membrane potential closer to the action potential threshold and thereby reducing the time required for ultrasound-induced depolarization. The calcium signaling cascade activation mechanism proposed by Yoo [22] could have been accelerated in this context: changes in membrane potential driven by the coupled electric field may have led calcium-activated sodium channels such as TRPM4 [37,38] to reach their opening threshold earlier, thus lowering the calcium influx required through mechanosensitive channels. Meanwhile, the dependence of latency shortening on PRF matched ED50 trends. The RS neuron kept a high shortening rate across a wide PRF range, whereas LTS exhibited a high shortening primarily at low frequency with a high duty cycle. This difference might have reflected channel kinetic properties. RS fast sodium channels activate and inactivate rapidly, allowing them to respond to electric-field-driven rapid depolarization even at high PRF. In contrast, the slow inactivation of LTS T-type calcium channels [39] limits full recovery at high PRF and thus constrains TMAS synergy. Overall, these distinctions highlight the importance of considering the electrophysiological characteristics of target neurons when designing TMAS parameters.

Like NICE/SONIC, the present framework links ultrasound-induced membrane-capacitance changes to nonlinear excitation thresholds [30,40]. Unlike these ultrasound-only models, the TMAS model additionally includes a Lorentz-force-related current density, which produced lower excitation thresholds and shorter first-spike latencies relative to TUS in the present simulations. Direct quantitative benchmarking will require matched neuronal models and stimulation parameters.

### 4.2. Dynamic Regulation of Firing Patterns and Activity-Associated Molecular Responses

The simulations showed that TMAS altered the distributions of Spike, Irregular-Burst, and Full-Burst states in the RS and LTS models (Figure 3 and Figure 4). This observation may underlie the superior neural encoding performance of TMAS [11] and aligns with recent animal studies demonstrating that TMAS restores abnormal neural oscillations [4,6,41,42]. Furthermore, this theoretical shift towards burst firing and enhanced spike synchrony perfectly aligns with our in vivo calcium imaging results; the population heatmaps revealed a much broader and more intensely synchronized neuronal activation pattern under TMAS compared to TUS. In the RS neuron, increasing the TUS dose drove a shift from spikes to bursts. Under TMAS, the addition of the coupled electric field significantly reduced the ultrasound dose required to elicit burst firing. At high PRF, the depolarization time window (τ) of a single ultrasound pulse was shorter than the inter-pulse interval (1 ms), which prevented depolarization summation and made spike triggering unstable. Under TMAS, however, the coupled electric field kept spike synchrony (Figure 4d). This suggested that the coupled electric field optimized firing patterns via two pathways. First, it accelerated inter-pulse depolarization accumulation so that a single pulse could induce multiple action potentials. Second, it prolonged the effective depolarization window, overcoming the constraints imposed by shortened inter-pulse intervals at high PRF. For LTS neuron, TMAS at PRF = 10 Hz required only a 3% increment in duty cycle to induce burst mode, while TUS required a higher dose. This further supported the potentiation effect of the electric field on low-threshold calcium channels. Notably, at high PRF, neither kind of stimulation produced bursting in LTS neurons. This restriction was due to rapid calcium-channel inactivation and strengthened repolarization by potassium currents [6], which defined the boundaries for the selective application of TMAS in high-frequency oscillatory networks.

Beyond acute electrophysiological modulation, our findings further suggest that the magneto-acoustic coupling effect is associated with activity-associated molecular responses. While calcium transients represent the immediate physiological response, the sustained influx of Ca2+ acts as a critical second messenger to trigger prolonged cellular alterations. This was supported by the significant upregulation of c-Fos in TMAS-treated neurons, indicating that the enhanced calcium influx can be effectively translated into immediate early gene transcription within the nucleus. Furthermore, the robust concurrent enhancement of MAP2 expression suggests potential cytoskeletal remodeling. Together, these molecular alterations suggest that TMAS does not merely evoke transient action potentials but may also support activity-dependent structural stabilization and functional refinement of neural networks at lower acoustic intensities than pure TUS.

### 4.3. Limitations and Future Perspectives

Several limitations should be acknowledged. First, although our computational framework incorporated dynamic membrane mechanics and calcium ion dynamics, it still simplifies the biophysical complexity of in vivo neural tissue. This single-compartment approximation does not resolve spatial current redistribution, conductivity interfaces, membrane area or neuronal morphology, extracellular return-current pathways, or orientation-dependent membrane polarization. Because the pressure-dependent scaling was derived from primary cortical-neuron calcium responses rather than from identified LTS interneurons, its application to the LTS T-type calcium current remains phenomenological and requires future cell-type-specific calibration. The broad pressure-duty-cycle sweep was designed to characterize the model response landscape and should not be interpreted as a recommended in vivo exposure range. Second, the molecular readouts used here, including c-Fos and MAP2, should be interpreted as indirect markers of recent cellular activity rather than definitive evidence of long-term functional plasticity. Third, the experimental preparations were not electrophysiologically identified as the RS or LTS neuronal types represented in the simulations, and fiber photometry measured population-level calcium signals rather than action potentials. The experiments therefore provide model-informed biological support but do not directly validate the predicted cell-type-specific firing thresholds, first-spike latencies, or firing-pattern transitions. In addition, the present study did not include separate sham-stimulation or static-magnetic-field-only groups and therefore could not fully distinguish the independent contributions of the static magnetic field and magneto-acoustic coupling. Fourth, while our in vitro and in vivo experiments provide computational and biological assessment of the enhanced neuromodulatory efficacy of TMAS, disease-specific therapeutic efficacy, long-term safety, and network- and behavior-level outcomes remain to be evaluated before clinical translation.

From a translational perspective, lower model-derived activation thresholds could reduce the required acoustic exposure, shorter response latency could improve temporal precision, and controllable spike-to-burst transitions could facilitate circuit-specific stimulation. These potential benefits require further validation in disease models using electrophysiological, behavioural, durability, and safety outcomes. Future work should formulate TMAS parameter selection as a constrained multi-objective optimization problem that jointly considers activation threshold, first-spike latency, acoustic exposure, firing-pattern control, and cell-type selectivity. Genetic algorithms, particle-swarm optimization, or Bayesian multi-objective optimization could be combined with experimental validation and safety constraints to identify Pareto-optimal stimulation parameters. Despite these limitations, the present findings indicate that TMAS expands the dynamic range of neuromodulation, improves energy efficiency, and offers enhanced spatiotemporal control, thereby opening new opportunities for probing neural circuits and developing precision neuromodulation strategies.

## 5. Conclusions

This study combined single-neuron modelling with in vitro and in vivo measurements to compare TMAS and TUS. Across the deterministic RS- and LTS-neuron parameter maps, inclusion of the modelled magneto-acoustic current reduced the model-derived ED50, shortened first-spike latency, and shifted response domains toward burst firing. Under two prespecified near-boundary conditions, TMAS also produced larger calcium-associated responses than TUS at matched nominal ultrasound parameters, together with increased c-Fos and MAP2 immunoreactivity. These findings support the potential of TMAS for dose-efficient neuromodulation and model-informed parameter selection. Further cell-type-specific, sham-controlled, and subject-specific acoustic–thermal studies are required before therapeutic translation.

## Data Availability

The raw experimental data and simulation code generated in this study are available from the corresponding author upon reasonable request.

## Supplementary Materials

The following supporting information can be downloaded at: https://www.mdpi.com/article/10.3390/brainsci16090973/s1, Supplementary Methods S1–S3, including the detailed computational model formulation, numerical settings, sensitivity and robustness analyses, and acoustic-exposure and thermal-safety estimation; Supplementary Results R1–R6; Supplementary Tables S1–S4, reporting model parameters, activation-threshold sensitivity, numerical convergence, latency inclusion, firing-pattern robustness, and probit goodness-of-fit; and Supplementary Figures S1–S4, showing calcium-current scaling sensitivity, conductivity sensitivity, thermal estimates, and population-level Fura-2 calcium quantification.
