# Supplementary material for: Computational–Experimental Characterization of Transcranial Magneto-Acoustic Stimulation for Dose-Efficient Neural Activation
Source: Brain Sci. 2026 Sep 14;16(9):973. doi: 10.3390/brainsci16090973 (PMC13604250; doi:10.3390/brainsci16090973)
Supplement: Supplementary file 1 [file brainsci-16-00973-s001.zip › brainsci-4507215-supplementary.pdf]

Supplementary Materials

Computational-Experimental Characterization of Transcranial Magneto-Acoustic Stimulation for Dose-Efficient Neural Activation

Ruxin Tan [1,†], Fangxuan Chu [2,†], Xin Wang [2], Xiaoqing Zhou [2], Ren Ma [2], Tao Yin [2], Haojun Fan [1,*] and Zhipeng Liu [2,3,*]

Supplementary Methods

S1. Computational Model and Numerical Implementation

S1.1. Membrane Mechanics

In the SONIC model, the nanoscale bilayer structures embedded within the plasma membrane undergo minute displacements (Z) in an ultrasonic field, leading to dynamic changes in the membrane capacitance (Cm). The relationship between the two can be expressed as:

$$\mathrm{C_m(z)} = \frac{\mathrm{C_{m0}}\Delta}{\mathrm{a^2}}\left[\mathrm{Z} + \frac{\mathrm{a^2 - Z^2 - Z\cdot\Delta}}{\mathrm{2Z}}\ln\left(\frac{\mathrm{2Z+\Delta}}{\Delta}\right)\right] \tag{1}$$

Periodic membrane capacitance oscillations near resonant structures can induce pronounced fluctuations in the membrane potential (Vm). The effect of such potential changes on neuronal activation is cell-type dependent and can be further computed using a modified Hodgkin–Huxley (HH) differential system. Therefore, local variations in membrane potential are associated not only with various ionic currents, each having specific reversal potentials and conductances, but also with capacitive displacement currents. The capacitive displacement current (Ic) induced by ultrasound can be expressed as:

$$\mathrm{I_c} = \mathrm{V_m}\frac{\mathrm{dC_m}}{\mathrm{dt}} \tag{2}$$

S1.2. Electrical Response

By introducing an update time step (here, Tup = 50 μs), the mechanical and electrical components of the model are decoupled, thereby reducing computational stiffness. To avoid directly operating on the rapidly oscillating Cm, the electrical part of the HH equations is temporally reformulated in terms of the smoother membrane charge Qm:

$$Q_m(t) = C_m(t)\cdot V_m(t) \tag{3}$$

The rapid oscillations of membrane capacitance are thus transformed into a smooth variation of membrane charge, which improves numerical stability. In this charge-based form, the membrane equation can be written as:

$$\frac{dQ_m}{dt} = -\sum I_{ion} + I_{stim} \tag{4}$$

Where Iion represents the ionic currents through various ion channels, and Istim is the externally applied stimulation

current.

S1.3. Ion Channel Model

In this study, regular spiking (RS) neurons and low-threshold spiking (LTS) neurons were selected as modeling targets. RS neurons are excitatory neurons, while LTS neurons are a common subtype of inhibitory interneurons and play a crucial role in maintaining the excitatory-inhibitory balance within neuronal circuits. The difference between the resting membrane potential and the firing threshold in LTS neurons is approximately 12 mV, which is significantly smaller than that of excitatory neurons or other types of inhibitory neurons. LTS neurons possess T-type calcium channels (low-voltage-activated calcium channels); therefore, under ultrasound stimulation, not only is the membrane capacitance affected, but the calcium current is also directly modulated.

The SONIC model is based on the intramembrane cavitation hypothesis. Ultrasound-induced calcium responses can involve mechanosensitive channels such as Piezo1, but an optical calcium-fluorescence response is not equivalent to a proportional change in T-type calcium current. Zhu et al. [24] reported a cortical regression of peak $\Delta F/F_0$ (%) = 1.132x + 0.5542, where x is acoustic pressure in MPa (six mice; tested cortical pressure range, approximately 0.06–0.25 MPa). The source article did not report R^2 for the cortical line.

The original code implementation used a pressure-dependent factor during ultrasound-on periods:

$$I_{ca}' = [1.32 * 10^{-6} * P(t) + 1.5542] I_{ca} \quad (5)$$

where P(t) is local acoustic pressure in Pa. This implemented factor is neither identical to nor a direct unit-consistent conversion of the published cortical fluorescence regression and is treated as a phenomenological scaling of the LTS T-type calcium current rather than a validated Piezo1 current or channel-gating law. The RS neuron model was constructed according to the literature, incorporating sodium ion channels, delayed rectifier potassium ion channels, M-type potassium ion channels, and leakage currents. In addition to these, the LTS inhibitory interneuron model also includes T-type calcium channels.

For completeness, the implemented charge-based neuronal equations are listed below. All maximal conductances and stimulation currents are expressed per unit membrane area.

$$\text{RS:} \quad dQ_m/dt = I_{stim} - 10^{-3}(I_{na} + I_k + I_m + I_l)$$

$$\text{LTS:} \quad dQ_m/dt = I_{stim} - 10^{-3}(I_{na} + I_k + I_m + I_t + I_l)$$

$$I_{na} = G_{na} m^3 h (V_{e}ff - E_{na}); \qquad I_k = G_k n^4 (V_{e}ff - E_k)$$

$$I_m = G_m p (V_{e}ff - E_k); \qquad I_l = G_l (V_{e}ff - E_l)$$

$$\text{LTS only:} \quad I_t = G_t s^2 u (V_{e}ff - ECa)$$

For each gate x ∈ {m, h, n, p, s, u}: $dx/dt = \alpha_x(Q_m,P) - [\alpha_x(Q_m,P) + \beta_x(Q_m,P)]x$

Here, Q_m is membrane charge density, $V_{e}ff$ is the effective membrane voltage recovered from the SONIC lookup tables, and P is local acoustic pressure. The α_x and β_x terms are pressure- and charge-dependent rates interpolated from the SONIC lookup tables rather than separate closed-form voltage-rate equations. During ultrasound-on periods, the LTS T-type current additionally contains the phenomenological pressure-dependent factor defined above.

S1.4. TMAS-Induced Current Density

In the TMAS simulations, the vibration velocity of induced charged particles under the influence of Lorentz force is determined by the ultrasonic acoustic pressure.

$$v = \frac{P}{\rho c} \tag{6}$$

where v is the vibration velocity of the charged particles, P is the ultrasound acoustic pressure, ρ is the density of biological tissue, and c is the speed of sound. The moving charged particles in a static magnetic field are subjected to a Lorentz force F.

$$F = qv \times B \tag{7}$$

The current density in tissue with electrical conductivity σ satisfies

$$J_{TMAS} = \frac{\sigma}{\rho c} BP \tag{8}$$

In the primary simulations, σ=0.50 S/m, B=0.3 T, ρ=1028 kg/m^3, and c=1515 m/s. The formulation assumes a locally plane acoustic wave and an orthogonal relationship between acoustic particle velocity and the magnetic field. The induced-current direction is defined by v × B; the model retains its magnitude and applies the positive scalar value as an inward depolarizing current density. Because no spatial electromagnetic or finite-element problem was solved, no tissue-interface or cellular-geometry boundary conditions were imposed.

S1.5 Initial Conditions, Solver Settings, and Convergence Assessment

The RS and LTS neurons were implemented as single-compartment models per unit membrane area; no explicit soma diameter, total membrane area, dendritic tree, or axonal geometry was introduced. Accordingly, conductances are reported in S/m^2, membrane capacitance in F/m^2, and applied currents as current densities.

Simulations were run in MATLAB R2022a using the variable-step, variable-order ode113 solver. The baseline absolute and relative tolerances were both 1×10^{-6}. The maximum solver step was the smaller of 25 μs and 0.1 times the shorter active-pulse duration (duty cycle divided by pulse-repetition frequency) for the ultrasound and electrical inputs. The SONIC mechanical-to-electrical update interval was 50 μs. The deterministic parameter-mapping grid used PRFs of 10, 100, and 1000 Hz, 100 linearly spaced duty cycles from 0.01 to 1.00, and 30 logarithmically spaced acoustic pressures from 10 to 600 kPa.

Initial membrane charge was Q_m,0 = C_m,0V_m,0, with C_m,0 = 0.01 F/m^2 and the cell-specific resting potentials listed in Supplementary Table S2. Each gating variable was initialized at its zero-pressure steady state, $x_0 = \alpha_x/(\alpha_x + \beta_x)$, evaluated at Q_m,0 using the SONIC lookup tables (0.5-MHz lookup condition).

A targeted convergence assessment was performed for eight representative RS/LTS and TUS/TMAS conditions spanning low and high PRF. Baseline results were compared with a stricter run using absolute and relative tolerances of 1×10^{-8} and one-half of the baseline maximum step. Activation status and firing-pattern category were unchanged in all eight comparisons. The maximum absolute firing-rate difference was 1 Hz (maximum relative difference, 1.5625%), and the maximum absolute first-spike-latency difference was 0.0637 ms (maximum relative difference, 0.2019%;

Supplementary Table S3). This targeted check evaluates numerical stability at representative conditions and does not constitute a complete rerun of every point in the ED50 parameter grid.

S2. Sensitivity and Robustness Analyses

S2.1 Activation-Threshold Sensitivity

The same deterministic firing-rate arrays used in the primary analysis were reclassified using activation criteria of 1, 2, 5, and 10 Hz. Firing rate was calculated as the number of action potentials occurring during the 1-s stimulation interval divided by the stimulation duration; thus, the 1-Hz criterion corresponds to at least one action potential during stimulation. For each threshold and PRF, the probit-derived ED50 was recalculated descriptively using the existing pressure–duty-cycle grid. No additional neuronal simulations were required.

S2.2 Calcium-Scaling Sensitivity

To assess whether the simulated LTS-neuron responses depended on the assumed linear pressure-dependent calcium modulation, we performed a targeted sensitivity analysis using three scaling assumptions.

Original linear factor: $\mathrm{M_{linear}(P) = 1.5542 + 1.32 \times 10^{-6}\,P}$.

Saturating factor: $\mathrm{M_{sat}(P) = 1 + 1.4778\,P/(58{,}642.6 + P)}$

No additional calcium modulation: $\mathrm{M_{none}(P) = 1}$.

where P is expressed in Pa. The saturating function was constrained to equal unity at zero pressure and to approximate the original linear function at approximately 41.04 and 600 kPa. The no-additional-modulation condition was included as a conservative limiting case.

Nine prespecified LTS-neuron conditions were evaluated. Six were representative firing conditions selected from the original response maps: PRF = 10 Hz, DC = 8%, P = 54.42 kPa; PRF = 10 Hz, DC = 20%, P = 127.0 kPa; PRF = 100 Hz, DC = 17%, P = 223.3 kPa; PRF = 100 Hz, DC = 54%, P = 257.2 kPa; PRF = 1000 Hz, DC = 9%, P = 41.04 kPa; and PRF = 1000 Hz, DC = 57%, P = 146.2 kPa. Three additional low-pressure conditions were located between the TUS and TMAS activation boundaries: PRF = 10 Hz, DC = 50%, P = 23.329 kPa; PRF = 100 Hz, DC = 55%, P = 23.329 kPa; and PRF = 1000 Hz, DC = 55%, P = 23.329 kPa.

Each condition was evaluated under both TUS and TMAS. The original linear-model results were reused, and 36 new simulations were performed for the saturating and no-additional-modulation assumptions. Because the condition at PRF = 100 Hz, DC = 17%, and P = 223.3 kPa was close to a Spike/Irregular-Burst classification boundary, eight

additional simulations were conducted at DC = 16% and 18% under the two alternative models. Firing-pattern category, firing rate, and first-spike latency were evaluated using the same definitions as in the primary analysis.

S2.3 Conductivity Sensitivity

The baseline tissue conductivity used in the TMAS model was 0.50 S/m, following the previously published TMAS neuronal-modelling study cited in the manuscript. A lower value of σ=0.33 S/m, adopted from the cited CT-based brain-tissue TMAS model, was tested as an alternative.

Four prespecified TMAS cases were examined, all using a 1-MHz carrier at PRF = 10 Hz: RS-A (P = 200 kPa, DC = 24%), RS-B (P = 200 kPa, DC = 35%), LTS-A (P = 54.42 kPa, DC = 8%), and LTS-B (P = 127 kPa, DC = 20%). Each case was simulated using the baseline tissue conductivity of 0.50 S/m and the alternative value of 0.33 S/m, yielding eight deterministic simulations in total. The corresponding TMAS current densities at sigma = 0.50 and 0.33 S/m were 0.019263 and 0.012713 A/m^2 for RS-A and RS-B, 0.005241 and 0.003459 A/m^2 for LTS-A, and 0.012232 and 0.008073 A/m^2 for LTS-B, respectively. Simulations lasted 2 s, with a 1-s stimulation train delivered from 0.5 to 1.5 s. The magnetic flux density, tissue density, and sound speed were held at 0.3 T, 1028 kg/m^3, and 1515 m/s, respectively; all other neuronal and numerical settings were identical between the two conductivity conditions. Firing-pattern category, firing rate, and first-spike latency were recorded.

S2.4 Latency Inclusion and Firing-Pattern Robustness

First-spike latency was compared only between identical PRF–duty-cycle–pressure combinations that generated at least one action potential under both TUS and TMAS. Parameter combinations were classified as co-triggered, TMAS-only, TUS-only, or inactive under both conditions. Only co-triggered combinations were included in the descriptive latency summaries; no inferential statistical tests were applied because the grid points represent deterministic model evaluations rather than independent replicates.

Firing-pattern robustness was assessed by reclassifying the existing simulation outputs using alternative definitions, including Full-Burst coverage thresholds of 90% and 80% of the pulse periods and a stricter burst definition requiring at least three spikes within a pulse period. Agreement with the original classification was calculated as the proportion of firing conditions retaining the same category. The descriptive fit of the probit models was additionally evaluated using the area under the receiver-operating-characteristic curve and McFadden's pseudo-(R2).

S3. Acoustic Exposure and Thermal Safety Estimation

S3.1. Acoustic Exposure Estimation

The reported peak acoustic pressure of 41.04 kPa was measured at the nominal focus under free-field conditions without an intervening skull. For both experimental parameter sets, the calculated $I_{SPPA}$was 56.14 mW/cm² and the mechanical index was approximately 0.041. At duty cycles of 55% and 66%, I_{SPTA} was 30.88 and 37.05 mW/cm², respectively. For the 10-s stimulation duration, the corresponding acoustic energies per unit area were 0.3088 and 0.3705 J/cm². Assuming an equivalent circular focal area with a diameter of 1.04 mm, the estimated focal energies were 2.62 and 3.15 mJ, respectively. No direct transcranial pressure measurement was performed; applying the approximately 18% pressure-amplitude attenuation reported for ex vivo mouse skull yielded an approximate dentate-gyrus pressure of 33.7 kPa. This value is a literature-based estimate rather than an in situ measurement.

S3.2. Thermal Safety Estimation

Temperature rise was estimated for the higher-exposure in vivo parameter set (P=41.04kPa, DC=66%, 1 MHz, 10 s) using a one-dimensional Pennes bioheat model containing a 0.5mm skull layer and a 10mm brain layer. The duty-cycle-averaged acoustic heat source was calculated as $Q_{US} = 2\alpha I_{SPPA}DC$. Joule heating associated with the modelled magneto-acoustic current was included as $Q_J = DC \times \frac{J_{TMAS}^2}{\delta}$. The 0.3T static magnetic field was not treated as a time-varying inductive-heating source.

Zero-flux outer boundaries were imposed, and the same local acoustic intensity was conservatively applied throughout the slab without accounting for reflection or upstream attenuation. The simulation used a spatial step of 0.0125 mm and a time step of 0.1 ms, comprising 10 s of stimulation followed by 10 s of cooling. Numerical convergence was evaluated using coarser spatial and temporal resolutions.

Supplementary Results

R1. Activation-Threshold Sensitivity

The activation-threshold sensitivity results are reported in Supplementary Table S1. Across the 1-, 2-, 5-, and 10-Hz criteria, the TMAS-associated reduction in model-derived ED50 remained between 71.4% and 76.3% for RS neurons. For LTS neurons, the reduction was 51.5–58.2% at 1 Hz, 40.6–48.7% at 2 Hz, 25.4–32.6% at 5 Hz, and 13.7–21.8% at 10

Hz. Thus, the direction of the TUS–TMAS difference was retained under every tested criterion, whereas its quantitative magnitude was more sensitive to the activation definition in the LTS model.

R2. Calcium-Scaling Sensitivity

The sensitivity-analysis results are summarized in Supplementary Fig. S1. Under the saturating assumption, 17 of the 18 TUS/TMAS conditions retained the firing-pattern category obtained with the original linear model. The maximum absolute changes in firing rate and first-spike latency were 4 Hz and 4.68 ms, respectively. The only category change occurred under TUS at PRF = 100 Hz, DC = 17%, and P = 223.3 kPa, where the response changed from Spike to Irregular-Burst and the firing rate changed from 68 to 71 Hz.

Under the no-additional-modulation assumption, 15 of the 18 conditions retained their original firing-pattern category. The largest firing-rate change occurred in the TMAS condition at PRF = 100 Hz, DC = 54%, and P = 257.2 kPa, where the firing rate decreased from 176 to 151 Hz. The observed category changes occurred near the 100-Hz Spike/Irregular-Burst boundary or represented a change between low-frequency Spike and Irregular-Burst labels.

Most importantly, TUS remained inactive and TMAS remained active at all three low-pressure near-boundary conditions under both alternative assumptions. At PRFs of 10, 100, and 1000 Hz, the corresponding TMAS firing rates were 7, 4, and 3 Hz under the saturating model and 4, 3, and 3 Hz under the no-additional-modulation condition, whereas the TUS firing rates remained 0 Hz. The additional analysis at PRF = 100 Hz and DC = 16%–18% showed that the isolated category changes were confined to a narrow classification boundary and did not represent a stable reversal of the relative TUS–TMAS response.

These results indicate that the assumed pressure-dependent calcium scaling affects quantitative firing rates, first-spike latencies, and the precise location of individual firing-pattern boundaries.

R3. Conductivity Sensitivity

Reducing conductivity from 0.50 to 0.33 S/m did not change the qualitative response category in any of the four paired comparisons(Supplementary Fig. S2). The firing rates changed from 72 to 60 Hz and from 142 to 122 Hz in the two RS cases, remained at 10 Hz in the LTS-A case, and changed from 67 to 63 Hz in the LTS-B case. First-spike latency increased by approximately 1.32–2.53 ms at the lower conductivity.

These results indicate that tissue conductivity affects the quantitative response magnitude and timing, but the principal firing patterns of the selected representative cases remain qualitatively robust.

R4. Latency Inclusion and Classification Robustness

Recruitment and conditional-latency counts are summarized in Supplementary Table S4A. Across the three PRFs, 4,638 of 9,000 RS parameter combinations and 6,860 of 9,000 LTS combinations generated at least one action potential under both TUS and TMAS and were included in the matched latency summaries. The remaining 4,362 RS and 2,140 LTS combinations were excluded from conditional latency because one or both stimulation modes did not fire. No TUS-only activation occurred; TMAS-only activation occurred at 954 RS and 718 LTS combinations. Across the matched co-triggered conditions, the mean TUS-minus-TMAS first-spike-latency difference was 41.40 ms for RS and 9.13 ms for LTS neurons. These are descriptive differences across deterministic parameter combinations rather than population-level statistical effects.

Classification robustness is summarized in Supplementary Table S4B. When Full-Burst coverage was relaxed from 100% to 90% or 80%, or when a burst-containing pulse window was required to contain at least three rather than two spikes, 91.7–96.3% of the 24,668 firing-condition labels were retained. Agreement ranged from 94.2% to 96.5% for RS and from 90.0% to 96.1% for LTS neurons, indicating that the overall pattern landscape was robust while a minority of labels near discrete category boundaries remained definition-sensitive.

R5. Thermal Safety Results

As shown in Supplementary Fig. S3a, the predicted maximum temperature rises were 0.0858 °C in skull and 0.0652 °C in brain, followed by gradual cooling after stimulation. Supplementary Fig. S3b shows the corresponding depth-dependent temperature distributions at the end of stimulation and at 10 s. Both peak values were substantially below the 2 °C level associated with nonsignificant thermal risk in the ITRUSST consensus. Grid refinement changed the predicted skull and brain peaks by approximately 0.002% and 0.47%, respectively.

The applied 0.3T magnetic field was static and was therefore not treated as a time-varying inductive-heating source. We nevertheless included resistive heating associated with the modeled magneto-acoustic current. The duty-cycle-averaged Joule heat source was 2.223×10^{-5} W/m3, compared with approximately 6.8×10^{4} W/m3 and 4.3×10^{3} W/m3 for acoustic heating in skull and brain, respectively (Supplementary Fig. S3c). Removing the Joule-heating term changed the predicted peak brain temperature by only 3.91×10^{-11}°C, indicating that current-associated heating was negligible within the assumptions of the model.

R6. In Vitro Calcium Quantification

Across three independent culture batches, the mean $\Delta F / F_0$ was higher in both TUS and TMAS groups than in the CON group. Under each matched parameter set, TMAS produced a greater calcium response than TUS (both ($p<0.05$); Supplementary Fig. S4). Relative to CON, the differences were ($p<0.05$) for TUS-1 and TUS-2, ($p<0.01$) for TMAS-1, and ($p<0.001$) for TMAS-2.

Supplementary Tables

Supplementary Table S1. Activation-threshold sensitivity of model-derived ED50

RS model

Threshold (Hz)	PRF (Hz)	TUS ED50	TMAS ED50	Reduction (%)
1	10	1314.29	375.32	71.44
1	100	2216.08	526.21	76.25
1	1000	2159.54	527.90	75.56
2	10	1332.82	375.99	71.79
2	100	2256.79	559.62	75.20
2	1000	2187.67	534.85	75.55
5	10	1443.48	408.87	71.67
5	100	2584.66	643.33	75.11
5	1000	2259.64	580.74	74.30
10	10	1992.98	553.22	72.24
10	100	3691.36	918.54	75.12
10	1000	2436.19	669.22	72.53

LTS model

Threshold (Hz)	PRF (Hz)	TUS ED50	TMAS ED50	Reduction (%)
1	10	93.16	38.94	58.20
1	100	94.48	45.85	51.47
1	1000	94.48	45.77	51.56
2	10	96.72	49.60	48.72
2	100	98.19	58.38	40.55
2	1000	99.15	58.66	40.84
5	10	106.13	71.51	32.63

Threshold (Hz)	PRF (Hz)	TUS ED50	TMAS ED50	Reduction (%)
5	100	109.09	81.44	25.35
5	1000	110.36	81.87	25.82
10	10	118.24	92.50	21.77
10	100	121.84	102.10	16.20
10	1000	121.87	105.23	13.66

Supplementary Table S2. RS and LTS single-neuron model parameters and numerical settings

A. Model and medium parameters

Category	Parameter (unit)	RS	LTS
Geometry	Formulation	Single compartment; per unit membrane area	Single compartment; per unit membrane area
Membrane	C_m,0 (F/m^2)	0.01	0.01
Membrane	V_m,0 (mV)	−71.9	−54
Na^+ current	G_{na} (S/m^2)	560	500
Na^+ current	E_{na} (mV)	50	50
K^+ current	G_k (S/m^2)	60	40
K^+ current	E_k (mV)	−90	−90
M current	G_m (S/m^2)	0.75	0.28
T-type Ca^{2+} current	G_t (S/m^2)	Not included	4
T-type Ca^{2+} current	ECa (mV)	Not included	120
Leak current	G_l (S/m^2)	0.205	0.19
Leak current	E_l (mV)	−70.3	−50
Medium	Sound speed (m/s)	1515	1515
Medium	Density (kg/m^3)	1028	1028
SONIC	Sonophore radius (nm)	32	32
SONIC	Temperature (K)	309.15	309.15

B. Numerical implementation and initialization

Item	Specification	Baseline setting	Convergence setting
Software/solver	MATLAB version and ODE solver	R2022a; ode113	R2022a; ode113
Tolerance	Absolute and relative tolerances	Both 1×10^{-6}	Both 1×10^{-8}
Maximum step	Adaptive upper bound	Smaller of 25 μs and 0.1 × the shorter active-pulse duration	One-half of baseline maximum step
Update interval	SONIC mechanical-to-electrical update	50 μs	50 μs
Initial charge	Q_m,0	C_m,0V_m,0	Same
Initial gates	x_0 for m, h, n, p, s, and u as applicable	$\alpha_x/(\alpha_x + \beta_x)$ at zero pressure and Q_m,0	Same

Supplementary Table S3. Targeted numerical convergence analysis of representative RS and LTS single-neuron conditions

Values in the firing-rate, latency, and pattern columns are shown as baseline/strict. Pressure is peak acoustic pressure. The strict profile used AbsTol = RelTol = 1×10^{-8} and one-half of the baseline maximum step.

Model	Stim.	PRF (Hz)	DC	P (kPa)	Firing rate (Hz)	Latency (ms)	Pattern
RS	TUS	10	0.24	200	10 / 10	24.3422 / 24.3407	Spike / Spike
RS	TMAS	10	0.24	200	72 / 72	11.5757 / 11.5748	Full-Burst / Full-Burst
RS	TUS	1000	0.42	127	64 / 63	73.8940 / 73.9050	Spike / Spike
RS	TMAS	1000	0.42	127	126 / 125	31.5556 / 31.6193	Spike / Spike
LTS	TUS	10	0.08	54.42	10 / 10	16.6927 / 16.6910	Spike / Spike
LTS	TMAS	10	0.08	54.42	10 / 10	12.2953 / 12.2934	Spike / Spike
LTS	TUS	1000	0.09	41.04	8 / 8	85.5996 / 85.5773	Spike / Spike
LTS	TMAS	1000	0.09	41.04	9 / 9	69.6897 / 69.6990	Spike / Spike

Across the eight comparisons, activation classification and firing-pattern category were unchanged. The maximum absolute differences were 1 Hz in firing rate and 0.0637 ms in first-spike latency.

Supplementary Table S4. Sensitivity of latency inclusion and firing-pattern definitions

A. Recruitment counts and conditions included in paired conditional-latency summaries

Model	PRF (Hz)	Co-triggered	TMAS only	TUS only	Neither	Excluded from paired latency
RS	10	1,625	289	0	1,086	1,375
RS	100	1,505	335	0	1,160	1,495
RS	1,000	1,508	330	0	1,162	1,492
LTS	10	2,290	269	0	441	710
LTS	100	2,285	224	0	491	715
LTS	1,000	2,285	225	0	490	715

B. Agreement of firing-pattern classifications under alternative definitions

Alternative definition	All firing conditions, n/N (%)	RS, n/N (%)	LTS, n/N (%)
Full-Burst coverage ≥90%	23,749/24,668 (96.27%)	9,870/10,230 (96.48%)	13,879/14,438 (96.13%)
Full-Burst coverage ≥80%	23,617/24,668 (95.74%)	9,843/10,230 (96.22%)	13,774/14,438 (95.40%)
Burst period ≥3 spikes	22,630/24,668 (91.74%)	9,639/10,230 (94.22%)	12,991/14,438 (89.98%)

C. Descriptive goodness-of-fit of the probit summaries

Model	Stim.	PRF (Hz)	Activated	Inactive	AUC	McFadden pseudo-R^2
RS	TUS	10	1,625	1,375	0.97759	0.51635
RS	TUS	100	1,505	1,495	0.96362	0.43725
RS	TUS	1,000	1,508	1,492	0.96518	0.44481
RS	TMAS	10	1,914	1,086	0.99166	0.69977
RS	TMAS	100	1,840	1,160	0.98706	0.63761
RS	TMAS	1,000	1,838	1,162	0.98714	0.63893
LTS	TUS	10	2,290	710	0.98580	0.77477
LTS	TUS	100	2,285	715	0.98612	0.77629
LTS	TUS	1,000	2,285	715	0.98612	0.77629
LTS	TMAS	10	2,559	441	0.99630	0.87317
LTS	TMAS	100	2,509	491	0.99464	0.84950
LTS	TMAS	1,000	2,510	490	0.99446	0.84704

Supplementary Figures

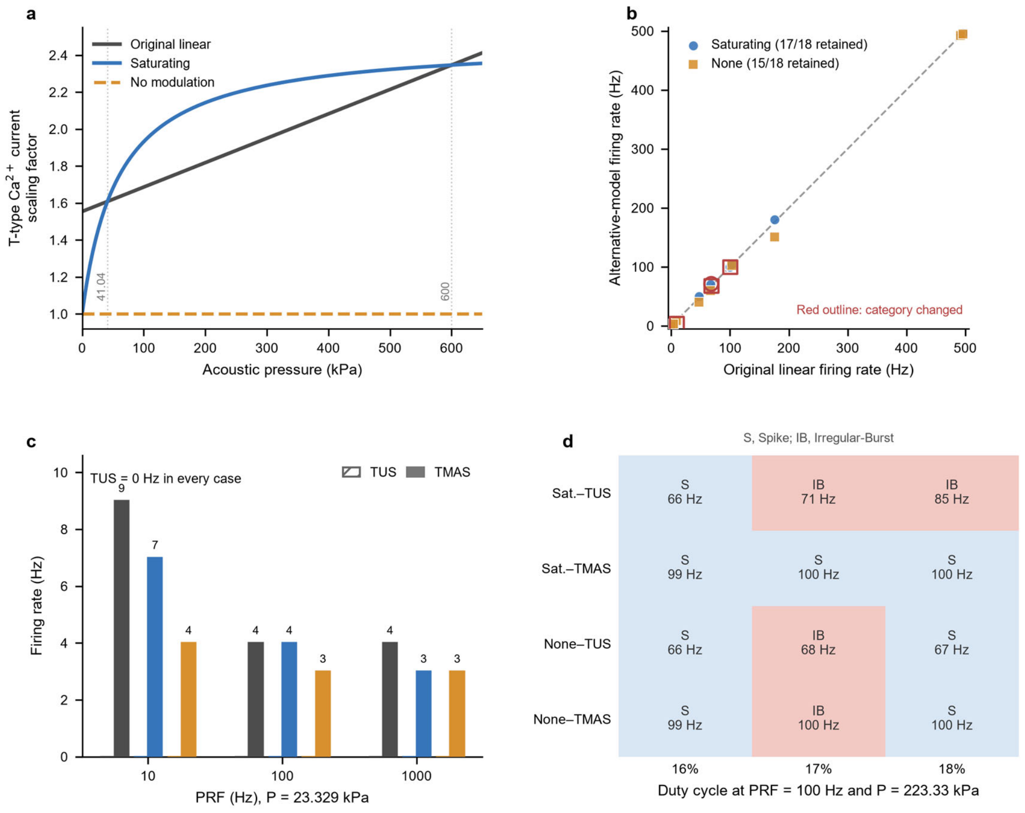

Supplementary Figure S1. Sensitivity of LTS-neuron responses to calcium-current scaling. (a) Linear, saturating, and no-additional-modulation assumptions. (b) Firing-rate comparison with the original linear model; red outlines indicate category changes. (c) Responses at three low-pressure near-boundary conditions, where TUS remained inactive and TMAS remained active. (d) Local assessment of the 100-Hz classification boundary at DC = 16%–18%. Results are from deterministic simulations.

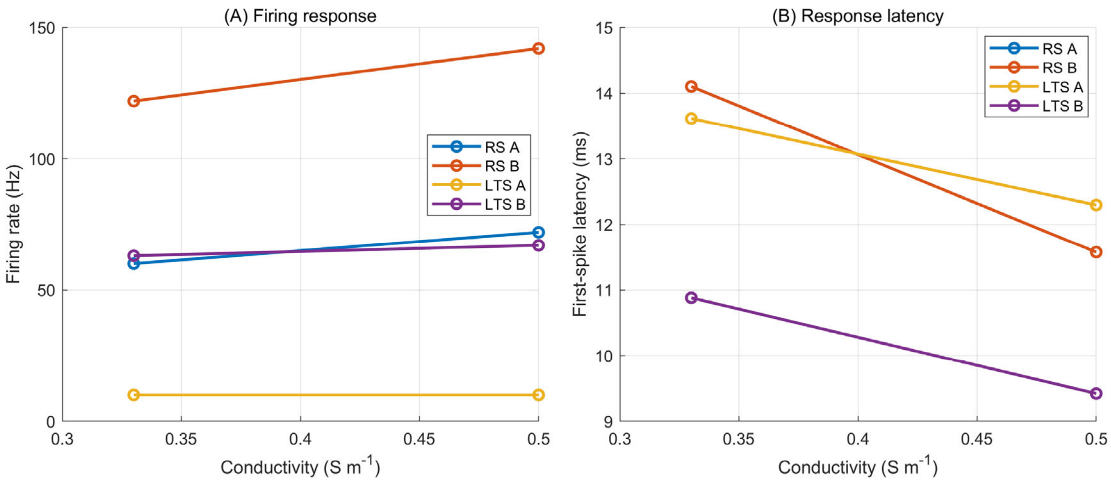

Supplementary Figure S2. Conductivity sensitivity of representative RS and LTS single-neuron simulations. (a) Firing rate and (b) first-spike latency at tissue conductivities of 0.33 and 0.50 S/m. All cases used a 1-MHz carrier and PRF = 10

Hz: RS-A, P = 200 kPa and DC = 24%; RS-B, P = 200 kPa and DC = 35%; LTS-A, P = 54.42 kPa and DC = 8%; and LTS-B, P = 127 kPa and DC = 20%. When conductivity was reduced from 0.50 to 0.33 S/m, firing rate changed from 72 to 60 Hz (RS-A), 142 to 122 Hz (RS-B), 10 to 10 Hz (LTS-A), and 67 to 63 Hz (LTS-B); first-spike latency changed from 11.5757 to 14.1023 ms (RS-A), 11.5757 to 14.1023 ms (RS-B), 12.2953 to 13.6146 ms (LTS-A), and 9.4205 to 10.8806 ms (LTS-B).

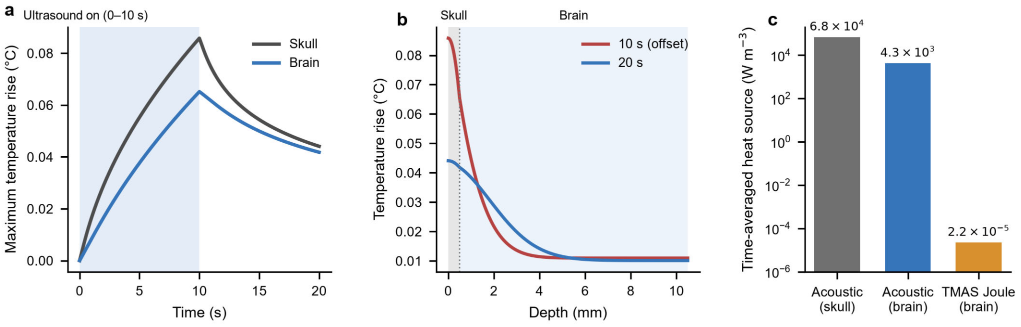

Supplementary Figure S3. Conservative thermal estimate for the higher-exposure in vivo TMAS condition. (a) Maximum predicted temperature rise in skull and brain during 10 s of stimulation and 10 s of cooling. The shaded region indicates ultrasound delivery. (b) Depth-dependent temperature-rise profiles at the end of stimulation (10 s) and after cooling (20 s); the dotted line indicates the skull–brain interface. (c) Time-averaged volumetric heat sources in skull and brain. Acoustic absorption dominated the thermal input, whereas magneto-acoustic Joule heating was negligible.

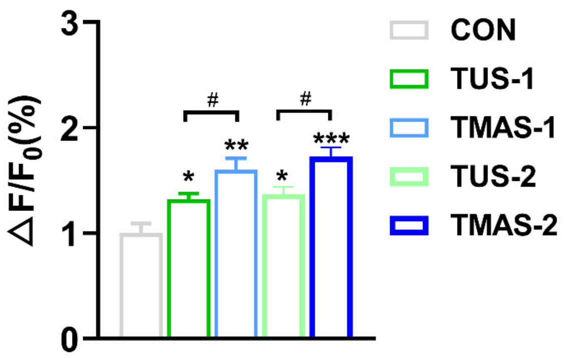

Supplementary Figure S4. Statistical analysis of $\Delta F/F_0$ (%) (n = 3). *$p < 0.05$, **$p < 0.01$, ***$p < 0.001$, vs the CON group; #$p < 0.05$ vs the TUS group.
